# Ceftolozane/tazobactam use and emergence of resistance: a 4-year analysis of antimicrobial susceptibility in *Pseudomonas aeruginosa* isolates in a tertiary hospital

**DOI:** 10.3389/fmicb.2025.1542491

**Published:** 2025-04-17

**Authors:** Alessandra Imeneo, Laura Campogiani, Pietro Vitale, Andrea Di Lorenzo, Grazia Alessio, Davide Natale Abate, Maria Grazia Celeste, Anna Altieri, Cartesio D’Agostini, Vincenzo Malagnino, Massimo Andreoni, Marco Iannetta, Loredana Sarmati

**Affiliations:** ^1^Department of System Medicine, University of Rome Tor Vergata, Rome, Italy; ^2^Infectious Disease Clinic, Policlinico Tor Vergata, Rome, Italy; ^3^Hospital Pharmacy, Policlinico Tor Vergata, Rome, Italy; ^4^Laboratory of Clinical Microbiology, Policlinico Tor Vergata, Rome, Italy; ^5^Department of Experimental Medicine, University of Rome Tor Vergata, Rome, Italy

**Keywords:** ceftolozane/tazobactam, *Pseudomonas aeruginosa*, fitness-cost, resistance, antibiotic susceptibility testing

## Abstract

**Background:**

Ceftolozane/tazobactam (C/T) was temporarily withdrawn from December 2020 to February 2022: this forced unavailability created the conditions to study how drug discontinuation might influence *Pseudomonas aeruginosa* (PA) resistance reversibility in a real-life setting.

**Methods:**

Clinically relevant PA isolates collected between January 1st 2019 and February 22nd 2023 with a C/T susceptibility test available were included. Changes in PA antibiotic susceptibility towards C/T and other antibiotics were examined in three different periods (period A, March–December 2019 and March–December 2020, C/T available; period B, March–December 2021, C/T withdrawn; period C, March–December 2022, C/T reintroduced), also considering the overall consumption rate through the Defined Daily Dose per 100 bed-days per year.

**Results:**

Seven hundred and fifty-one PA isolates were included. A statistically significant reduction of C/T resistance rate was observed when C/T became unavailable, followed by a subsequent increase with its reintroduction (period A 25.1% vs. period B 5.3% vs. period C 10.0%, *p* < 0.001). A concomitant reduction of resistance rates towards other antibiotics was recorded, consistent with antibiotic consumptions and antimicrobial stewardship programs implementation. A subgroup of 22 patients presented a C/T-resistant isolate after a previous susceptible one; only 4 patients had received a prior C/T treatment.

**Conclusion:**

The unavailability of C/T created the conditions to analyze the practical application of the theory of fitness cost to maintain resistance. A subsequent increase after a first reduction in C/T resistance rate was observed, probably due to persistence of resistant isolates and antibiotic selective pressure. Continuous monitoring of antibiotic use and evolving resistance is essential.

## 1 Introduction

In recent years, the worldwide spread of multidrug-resistant bacteria (MDR) has become a public health threat. Along with the constant search for new antibiotics, a strategy is to discontinue the use of antibiotics against which resistance is widespread. This approach is based on the theory that the maintenance of resistance mechanisms requires a high fitness cost and so susceptible bacteria have greater replicative advantages than resistant bacteria, in the absence of the drug (Andersson and Hughes, 2010). However, resistance reversibility has been considered too slow to achieve and difficult to maintain (Baym et al., 2016; Melnyk et al., 2015; De Gelder et al., 2004), hence this approach is not used in clinical practice.

Together with *Enterobacterales* and *Acinetobacter baumanni*, *Pseudomonas aeruginosa* is recognized by the World Health Organization as a high-priority pathogen for which the development of new drugs is warranted (Tacconelli et al., 2018). *P. aeruginosa* has one of the largest bacterial genomes and is frequently resistant to antibiotics, due to several mechanisms: intrinsic resistance, spontaneous chromosomal mutations, horizontal gene acquisition within integrons and mobile genetic elements. The main intrinsic resistance strategies are: (1) a low outer membrane permeability, responsible for imipenem resistance, (2) the production of AmpC, which confer resistance to penicillins, cephalosporins and monobactams, (3) the production of efflux pumps. Specific resistance in DNA gyrase and topoisomerase are responsible for fluoroquinolone resistance. Finally, resistance to carbapenem results from: (1) chromosomal mutations; (2) metallo-beta-lactamases (MBL) production (such as VIM [Verona integron encoded], NDM [New Delhi MBL], IMP [imipenemase]); (3) serine-carbapenemases (such as KPC [*Klebsiella pneumoniae* carpabenemase]-2 and GES[Guyana Extended-Spectrum]-variants) acquisition (Botelho et al., 2019; Berrazeg et al., 2015; Horcajada et al., 2019). In this context, a valuable treatment option is eftolozane/tazobactam (C/T, Zerbaxa®), a combination of a novel oxymino-cephalosporin (ceftolozane) with a *β*-lactamase inhibitor (tazobactam) in a fixed 2:1 ratio, which has been approved for the treatment of complicated intra-abdominal infections (cIAI), complicated urinary tract infections (cUTI) and hospital-acquired pneumonia (HAP) caused by gram-negative bacteria. It showed a promising activity towards *P. aeruginosa,* due to a relative stability against the three most common mechanisms of beta-lactam resistance. Ceftolozane indeed showed high affinity for the essential penicillin-binding-protein of *P. aeruginosa* (e.g., PBP1b, PBP1c, PBP2 and PBP3), stability towards the chromosomal AmpC *β*-lactamase and to the Mex efflux pumps (Giacobbe et al., 2018; Pogue et al., 2020).

However, soon after its introduction, several reports of C/T resistance were described, emphasizing the importance of understanding its resistance mechanisms. First, *in vitro* studies have shown that the accumulation of several mutations, including those responsible for the overexpression and the structural modification of AmpC, can lead to C/T resistance development (Cabot et al., 2014). In addition, several cases of strains resistant to C/T, due to horizontally acquisition of ESBLs and/or carbapenemases, were reported (Fraile-Ribot et al., 2018; Fournier et al., 2021; Mojica et al., 2022; Teo et al., 2021; Del Barrio-Tofiño et al., 2017). Interestingly, patients treated with C/T were less likely to develop resistance than those treated with other *β*-lactams (Shah et al., 2025). In Europe and USA, the incidence of resistance rate to C/T varies between 3–10% and 22–32% in strains that are also co-resistant to most β-lactams (Karlowsky et al., 2024; Fournier et al., 2021; Del Barrio-Tofiño et al., 2017). Italian surveillance studies report a C/T resistance rate of 4–20% among *P. aeruginosa* strains and 15–32% among MDR *P. aeruginosa* strains (Bianco et al., 2022; Valzano et al., 2024; De Pascale et al., 2025).

C/T was approved by the US Food and Drug Administration (FDA) in 2014 and by the European Medicines Agency (EMA) in 2015 and became available in Italy in November 2016. Due to *Ralstonia pickettii* contamination of a limited number of Zerbaxa® batches, the drug was temporarily withdrawn from the market and was no longer available from December 2020 to February 2022 (AIFA, n.d.). This forced interruption of C/T distribution created the conditions to study how drug discontinuation might influence pathogens’ resistance patterns and resistance reversibility in a *real-life* setting. This study aimed at evaluating the resistance rate of *P. aeruginosa* towards C/T in relation to the availability of the antibiotic trying to further characterize the role of drug selective pressure on resistance acquisition and maintenance.

## 2 Materials and methods

This is a retrospective observational study performed at the University Hospital Policlinico Tor Vergata of Rome, Italy, including isolates of *Pseudomonas aeruginosa* collected from January 1st 2019 to February 22nd 2023.

A list of *P. aeruginosa* isolates with phenotypic antibiogram susceptibility tests available (including C/T) collected from any type of microbiological sample, was derived from the Hospital Microbiology Laboratory. Considering the evaluation of C/T resistance rate as the main goal of the study, all strains without antimicrobial susceptibility tests to C/T were excluded. Secondarily, we analyzed the resistance rate to other tested antimicrobials to compare the resistance trend to that of C/T. The antimicrobial susceptibility tests were performed with an automated Vitek-2 system or microdilution. To evaluate the production of some carbapenemases, an immunochromatographic assay NG CARBA® was also performed. The minimum inhibitory concentrations (MICs) were interpreted according to the most up-to-date European Committee on Antimicrobial Susceptibility Testing (EUCAST) clinical breakpoints available at the time of the test (in detail, EUCAST Clinical Breakpoint Tables v. 9.0 were consulted for strains collected in 2019, v. 10.0 in 2020, v. 11.0 in 2021, v. 12.0 in 2022, v. 13.1 in 2023). In particular, EUCAST Breakpoints define C/T as “*resistant*” if MIC > 4, otherwise is “*susceptible*”; moreover, dosages of 1 g ceftolozane + 0.5 g tazobactam are recommended for intrabdominal and UTI infections, while higher dose of 2 g ceftolozane + 1 g tazobactam very three hour is recommended for hospital acquired pneumonia. In our analysis, isolates were defined as *susceptible (S)* if presenting MICs defined as susceptible or intermediate according to the EUCAST breakpoints, or *resistant (R)*, in the remaining cases. In the statistical analysis, resistance to an antibiotic class is defined by the resistance of at least one antibiotic belonging to the class (e.g., the carbapenem resistance is defined by the resistance to meropenem and/or imipenem/cilastatin). In the presence of MICs ≥ *[n]*, these were considered as *[n]*, in the statistical analysis.

For a better selection of the isolates associated with infectious episodes, our analysis included:

- Isolates collected from blood, respiratory samples, urine, cerebrospinal fluid, pleural and peritoneal aspirates, central venous catheter (CVC) tip;
- Isolates with C/T susceptibility test available.

Isolates were excluded if no C/T susceptibility test was reported or if derived from swabs (e.g., rectal, skin), due to the inability to distinguish contamination/colonization from infection.

Only the first isolate for each patient was included; if multiple isolates from the same patient were available, a cut-off interval of 5 months was adopted to include subsequent isolates in the study.

Since Zerbaxa® was no longer available from the end of December 2020 to mid-February 2022, to make the study periods homogeneous, isolates collected in January and February of each year were excluded from the study. Therefore, based on the sample collection date, 3 periods of included isolates were identified:

- Period A: from March to December 2019 and from March to December 2020, when C/T was available;
- Period B: from March to December 2021, when C/T was no more available, following its market withdrawal;
- Period C: from March to December 2022, when C/T was available again.

From the overall list of *P. aeruginosa* isolates, further analysis was performed to identify a subgroup of patients who had a C/T-susceptible isolate followed by a C/T-resistant isolate within the same hospitalization and within 3 months.

To evaluate the influence of the consumption of other antibiotics on the resistance rates of *P. aeruginosa*, the overall consumption of each antibiotic in our hospital was estimated through the Defined Daily Dose (DDD) per 100 bed-days per year, calculated using the average daily dose for the main indication in adults of each antibiotic and the occupancy index of beds, corresponding to the percentage ratio between hospital days actually used by patients and those theoretically available.

### 2.1 Statistical analysis

All data were analyzed using JASP (Version 0.18.3). Categorical variables are presented as absolute frequency and percentages (%), while quantitative variables are presented as medians and interquartile ranges (IQR). Differences between groups were assessed with the two-tailed Chi-square test for categorical data and the Kruskal–Wallis test for quantitative data. The Dunn-Bonferroni post-hoc test and the measure of effect size (η^2^) were applied to the analysis of variance (ANOVA). For all the tests, the level of statistical significance was <0.05.

## 3 Results

**Figure 1:**
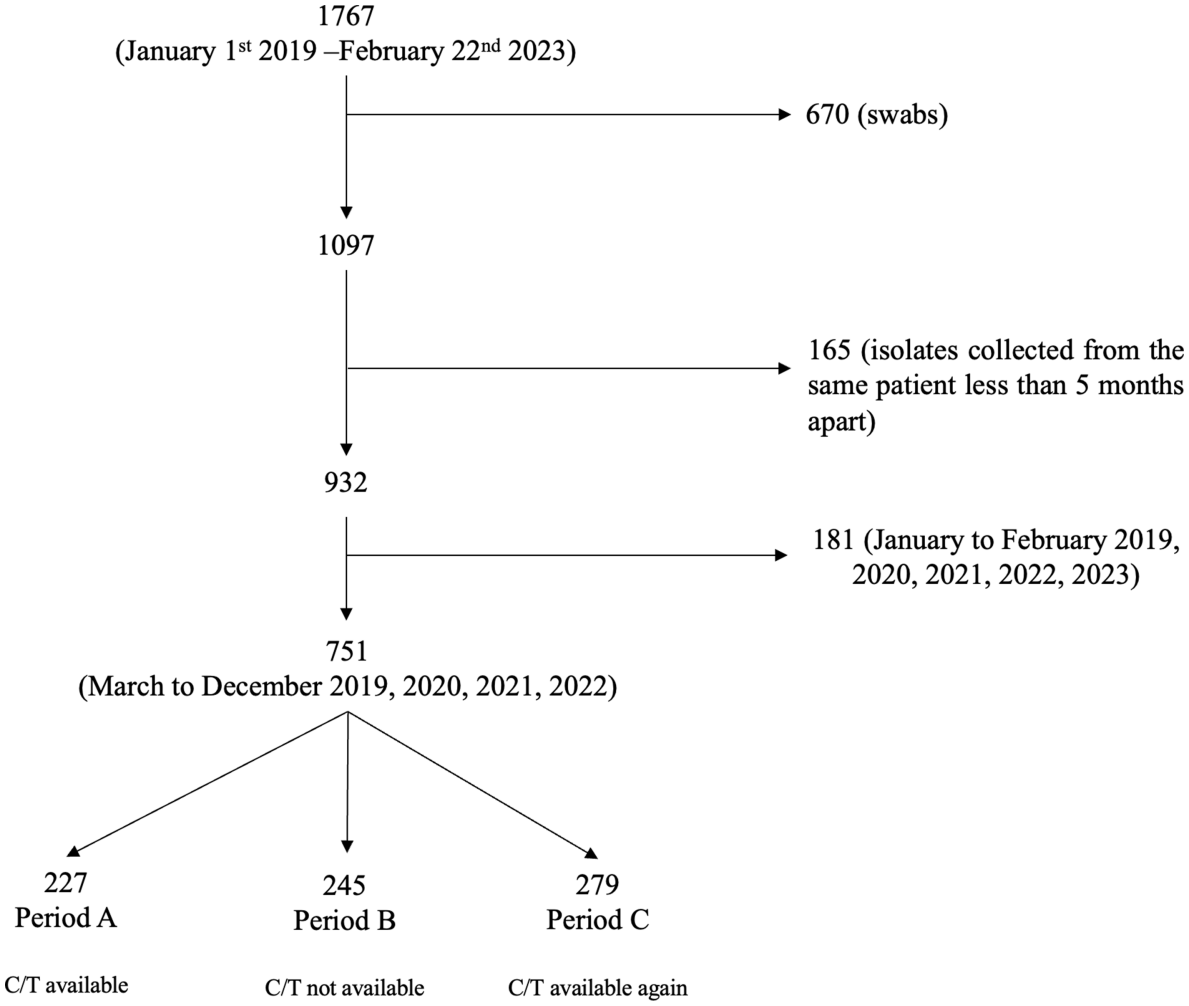
Flow chart of the selection process of *Pseudomonas aeruginosa* isolates. CIT, ceftolozane/tazobactam.

A total of 1767 *P. aeruginosa* isolates, collected between January 1st 2019 and February 22nd 2023, were evaluated. Due to the impossibility of distinguishing contamination/colonization from infection, 670 isolates derived from swabs were excluded; 165 isolates collected from the same patient less than 5 months apart were excluded. Furthermore, to include homogeneous months/year period in the analysis, 181 of the 932 isolates, collected between January to February of each year (2019, 2020, 2021, 2022, 2023), were excluded, resulting in a final population of 751 isolates (Figure 1).

Most of the isolates were collected from urine (42.2%) and respiratory samples (38.0%); no significant difference was observed in the biological source of the isolates in the three study period, except for urinary samples, prevalently collected during period B (period A 37% *Vs* period B 49% *Vs* period C 40.5%, *p*-value 0.024).

**Figure 2:**
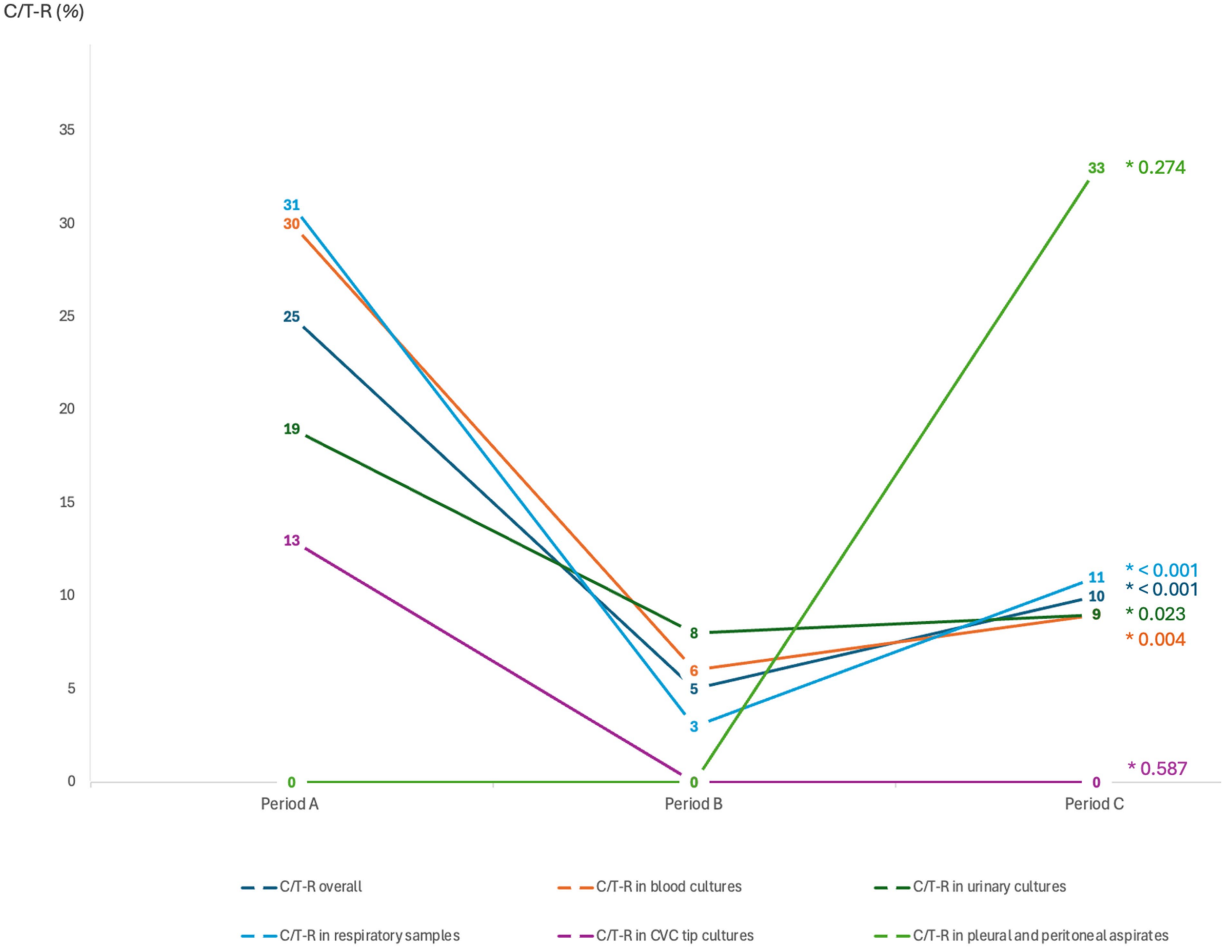
Distribution of the ceftolozane/tazobactam-resistant isolates (C/T-R), overall and after stratification according to the microbiological sample types. Categorical variables are presented as percentages (0/0). Differences between groups were assessed with the two-tailed Chi^2^ test for categorical variables (*p-value). Period A: March–December 2019 and March–December 2020 (CIT available); Period B: MarchDecember 2021 (C/T no more available); Period C: March–December 2022 (C/T available again).

The overall number of *P. aeruginosa* isolates resistant to C/T (C/T-R) was 98 (13%). A significantly higher number of resistant isolates was collected in period A (March–December 2019 and March–December 2020) (25.1%), when C/T was available, compared to period B (March–December 2021) (5.3%), when C/T was not available, followed by a significant raise of C/T resistant isolates in period C (March–December 2022) (10%) when C/T was available again (*p*-value <0.001) (Table 1). C/T-resistant *P. aeruginosa* isolates were also stratified according to the microbiological sample type (Figure 2). A significant change of C/T-R through the 3 study periods was observed, mainly for respiratory samples (period A 30.6% vs. period B 2.5% vs. period C 11.2%, *p* < 0.001), blood cultures and urine cultures (*p*-value 0.04 and *p*-value 0.023 respectively) while no differences were recorded for CVC tip cultures and pleural or peritoneal aspirates, probably due to the limited number of samples included.

**Table 1:** Antibiotics resistance rates in the different study periods.

	Period A (March–December 2019 and 2020) C/T available *227 isolates*	Period B (March–December 2021) C/T not available *245 isolates*	Period C (March–December 2022) C/T available *279 isolates*	*p*-value
**C/T-R**	**57 (25.1%)**	**13 (5.3%)**	**28 (10.0%)**	<0.001
TZP-R	86 (38.1%)	52 (21.4%)	78 (28.5%)	<0.001
MEM-R	67 (29.6%)	20 (8.2%)	31 (11.1%)	<0.001
IPM-R	78 (38.6%)	34 (14.5%)	57 (21.0%)	<0.001
CZA-R	42 (18.5%)	12 (4.9%)	26 (9.3%)	<0.001
ATM-R	32 (23.7%)	7 (13.2%)	4 (10.5%)	0.088
CAZ-R	75 (33.0%)	43 (17.6%)	52 (18.8%)	<0.001
FEP-R	63 (27.8%)	31 (12.7%)	42 (15.1%)	<0.001
AMK-R	22 (9.7%)	10 (4.1%)	18 (6.5%)	0.050
CST-R	10 (4.4%)	6 (2.4%)	2 (0.7%)	0.026
CIP-R	98 (43.2%)	45 (18.4%)	60 (21.7%)	<0.001
C/T-R, CZA-R, carbapenem-S	1 (0.4%)	1 (0.4%)	3 (1.1%)	0.569
C/T-R, CZA-R, carbapenem-R	39 (17.2%)	10 (4.1%)	18 (6.5%)	<0.001
C/T-R, CZA-S, carbapenem-R	9 (4.0%)	0 (0%)	3 (1.1%)	0.002
DTR	11 (4.8%)	2 (0.8%)	1 (0.4%)	0.001

Categorical variables are presented as percentages (%). Differences between groups were assessed using the two-tailed Chi^2^ test for categorical variables. C/T, ceftolozane/tazobactam; TZP, piperacillin/tazobactam, MEM, meropenem; IMP, imipenem/cilastatin; CZA, ceftazidime/avibactam; ATM, aztreonam; CAZ, ceftazidime; FEP, cefepime; AMK, amikacin; CST, colistin; CIP, ciprofloxacin; DTR, difficult-to-treat resistance (non-susceptibility to piperacillin/tazobactam, ceftazidime, cefepime, aztreonam, meropenem, imipenem/cilastatin, ciprofloxacin).C/T resistance rates in the three study periods are highlighted in bold values.

**Figure 3:**
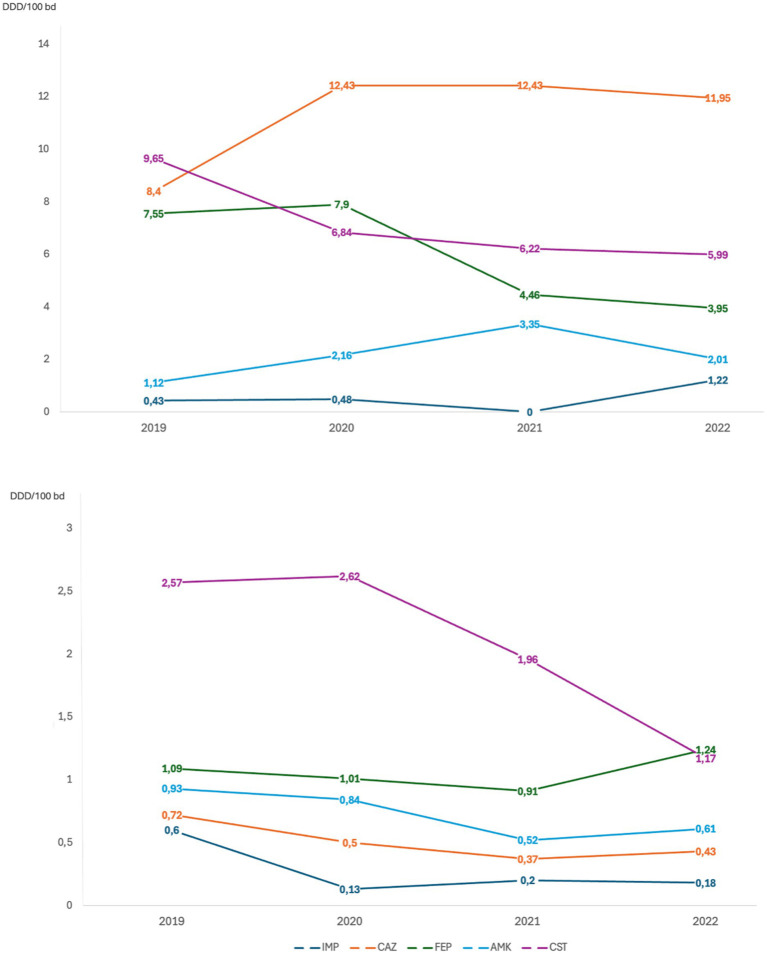
Antibiotic consumption per year evaluated through Defined Daily Dose per 100 bed-days per year (DDD/100 bd). CIT, ceftolozane/tazobactam; TZP, piperacillin/tazobactam; MEM, meropenem; CZA, ceftazidime/avibactam; CIP, ciprofloxacin; IMP, imipenem/cilastatin; CAZ, ceftazidime; FEP, cefepime; AMK, amikacin; CST, colistin.

To better understand if the changes observed in antimicrobial susceptibility rates were linked to a reduction in the use of different antibiotics, the consumption rates of C/T and other antibiotic molecules in the different study periods were evaluated, reported as DDD per 100 bed-days per year (Figure 3). During 2021, when C/T was no longer available we observed an increase in ceftazidime/avibactam consumption, followed by a new increase in C/T use during 2022, after the drug became available again (0.4 before withdrawal vs. 1.2 after reintroduction). In 2021 a decrease in the overall consumption rates of other molecules was also observed, probably related to the beginning of an antimicrobial stewardship program in our hospital. This was particularly noticeable for ciprofloxacin and imipenem, from 2020, and for meropenem and colistin, from 2021. Conversely, the consumption of piperacillin/tazobactam remained stably high.

To evaluate whether the emergence of *P. aeruginosa* C/T-R isolates was related to cross-reaction with other molecules, susceptibility trends to piperacillin/tazobactam, meropenem, imipenem/cilastatin, ceftazidime/avibactam, aztreonam, ceftazidime, amikacin, colistin, ciprofloxacin were also investigated (Table 1). For all the 751 *P. aeruginosa* isolates, a trend similar to C/T was found in all molecules studied, with a significant reduction of resistance in Period B (2021) and a stability or a slight increase in Period C (2022). The same trend was also observed for the 15 difficult-to-treat resistance (DTR) isolates, defined by non-susceptibility to all first-line agents (piperacillin/tazobactam, ceftazidime, cefepime, aztreonam, meropenem, imipenem/cilastatin and ciprofloxacin); all were susceptible to colistin, notably. Among the three periods analyzed, we observed 182 (24%) strains resistant to carbapenem (meropenem and/or imipenem). Among these, 79 (11%) were resistant to C/T also; 12 (2%) of these maintained susceptibility to ceftazidime/avibactam. Finally, 67 (9%) isolates showed resistance to carbapenem and both C/T and ceftazidime/avibactam, with isolation of metallo-*β*-lactamase VIM in 37 (55%) strains or IMP in 1 strain (1%).

**Figure 4:**
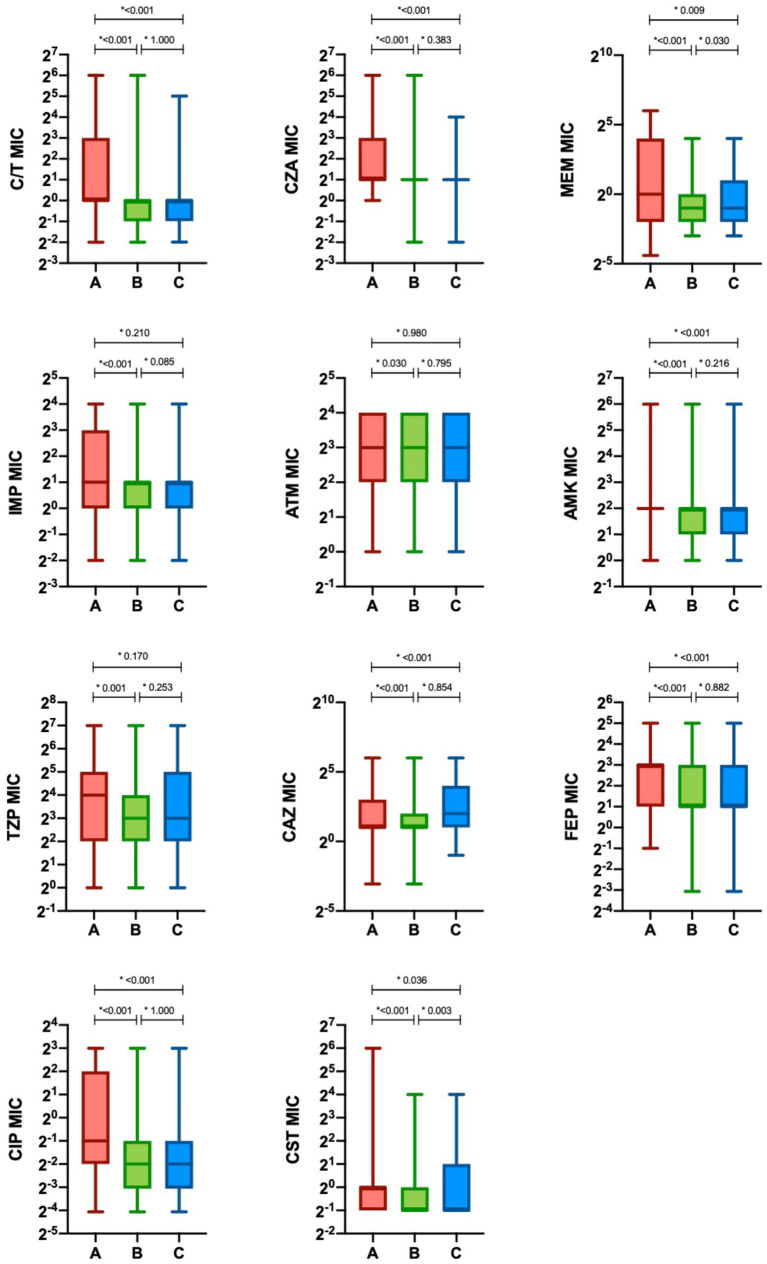
Minimum inhibitory concentration (MIC) of antimicrobials in the different study period. The minimum inhibitory concentrations (MIC) of antimicrobials are presented as median (interquartile range [IQR]) (boxes). Whiskers represent min-max range. Differences between groups were assessed with the Kruskal—Wallis test and the Dunn-Bonferroni post-hoc test (*p-value). CIT, ceftolozane/tazobactam; CZA, ceftazidime/avibactam; MEM, meropenem; IMP, imipenem/cilastatin; ATM, aztreonam; AMK, amikacin; TZP, piperacillin/tazobactam; CAZ, ceftazidime; FEP, cefepime; CIP, ciprofloxacin; CST, colistin; Period A: March–December 2019 and March–December 2020 (C/T available); Period B: March–December 2021 (C/T no more available); Period C: March–December 2022 (C/T available again).

To better characterize the sensitivity of *P. aeruginosa* strains towards different antibiotics, an analysis of the MIC trends of isolates in the study periods A, B and C was performed (Figure 4; Table 2). Except for aztreonam, for all the antimicrobials evaluated the highest MICs values were observed in Period A, followed by a subsequent and significant shift toward lower median values. A small effect size was observed due to the limited number of samples represented in our study.

**Table 2:** Minimum inhibitory concentration (MIC) of antimicrobials in the different study periods.

	Period A (March–December 2019 and 2020) C/T available *227 isolates*	Period B (March–December 2021) C/T not available *245 isolates*	Period C (March–December 2022) C/T available *279 isolates*	*p*-value	Effect size	Post-hoc analysis
C/T	1.0 [1.0–6.0]	1.0 [0.5–1.0]	1.0 [0.5–1.0]	<0.001	0.026	A-B: <0.001 A-C: <0.001 B-C: 1.000
TZP	16.0 [4.0–32.0]	8.0 [4.0–16.0]	8.0 [4.0–32.0]	0.002	0.014	A-B: 0.001 A-C: 0.170 B-C: 0.253
MEM	1.0 [0.25–16.0]	0.5 [0.25–1.0]	0.5 [0.25–2.0]	<0.001	0.076	A-B: <0.001 A-C: 0.009 B-C: 0.030
IPM	2.0 [1.0–8.0]	2.0 [1.0–2.0]	2.0 [1.0–2.0]	<0.001	0.017	A-B: <0.001 A-C: 0.210 B-C: 0.085
CZA	2.0 [2.0–8.0]	2.0 [2.0–2.0]	2.0 [2.0–2.0]	<0.001	0.032	A-B: <0.001 A-C: <0.001 B-C: 0.383
ATM	8.0 [4.0–16.0]	8.0 [4.0–16.0]	8.0 [5.0–16.0]	0.034	0.031	A-B: 0.030 A-C: 0.980 B-C: 0.795
CAZ	4.0 [2.0–16.0]	2.0 [2.0–4.0]	2.0 [2.0–8.0]	<0.001	0.020	A-B: <0.001 A-C: <0.001 B-C: 0.854
FEP	8.0 [2.0–8.0]	2.0 [2.0–8.0]	2.0 [2.0–8.0]	<0.001	0.014	A-B: <0.001 A-C: <0.001 A-C: 0.882
AMK	4.0 [4.0–4.0]	4.0 [2.0–4.0]	4.0 [2.0–4.0]	<0.001	0.005	A-B: <0.001 A-C: <0.001 B-C: 0.216
CST	1.0 [0.5–1.0]	0.5 [0.5–1.0]	0.5 [0.5–2.0]	<0.001	0.005	A-B: <0.001 A-C: 0.036 B-C: 0.003
CIP	0.5 [0.25–4.0]	0.25 [0.12–0.5]	0.25 [0.12–0.5]	<0.001	0.060	A-B: <0.001 A-C: <0.001 B-C: 1.000

The minimum inhibitory concentrations (MIC) of antimicrobials are presented as median (interquartile range [IQR]). Differences between groups were assessed with the Kruskal–Wallis test, the Dunn post-hoc test with the Bonferroni correction and the measure of effect size (η^2^) for quantitative data. C/T, ceftolozane/tazobactam; TZP, piperacillin/tazobactam, MEM, meropenem; IMP, imipenem/cilastatin; CZA, ceftazidime/avibactam; ATM, aztreonam; CAZ, ceftazidime; FEP, cefepime; AMK, amikacin; CST, colistin; CIP, ciprofloxacin.

Finally, a subgroup of 22 patients that had a C/T-resistant *P. aeruginosa* isolated after a previous C/T-susceptible one were analyzed, specifically, 5 patients during Period A, 8 patients during Period B and 9 patients during Period C (Table 3). These patients were mainly hospitalized in the Intensive Care Unit (12, 54.5%), most of them received antibiotic therapy between the two *P. aeruginosa* isolates, predominantly with ceftazidime/avibactam (12, 54.5%), colistin (11, 50.0%) and carbapenem (10, 45.5%). Only 4 patients (18.2%) received prior treatment with C/T monotherapy and 3 of them were treated with C/T high dosage because of hospital-acquired pneumonia (HAP). The median time between the isolation of C/T-R *P. aeruginosa* in a patient with a previous C/T-susceptible isolate was 23.5 [15.0–31.0] days.

**Table 3:** Characteristics of the patients with a C/T resistant isolate (C/T-R) after a first C/T susceptible isolate (C/T-S).

n°	Sex	Age	Period	Days between C/T-S and C/T-R isolates	Previous C/T treatment	Other previous treatment	Sample	Ward	C/T MIC (μg/mL)
1	M	50	2019 (A)	23		MEM	Blood	SD	≤1
							Blood	SD	8
2	F	64	2020 (A)	13		MEM, CZA	Respiratory	ER	1
							Respiratory	ER	≥32
3	M	67	2020 (A)	24	1,5 g every 3 h	CST, FEP, C/T, MEM, CZA	Respiratory	ER	1
							Respiratory	ICU	8
4	M	76	2020 (A)	25		TGC, CST, MEM	Respiratory	MD	1
							Urine	MD	>8
5	M	52	2020 (A)	5		NA	Blood	MD	≤1
							Blood	MD	>8
6	M	70	2021 (B)	68		MEM, CZA, CST, TGC	Blood	ICU	≤1
							Respiratory	ICU	8
7	F	65	2021 (B)	68		CZA, FEP, CST, TGC	Respiratory	ICU	≤1
							Respiratory	ICU	≥32
8	M	43	2021 (B)	34		CZA, MEM	Urine	ICU	0.5
							Respiratory	ICU	≥32
9	M	77	2021 (B)	15		TZP, CZA	Urine	ICU	≤1
							Respiratory	ICU	8
10	M	74	2021 (B)	31		CST, CZA, TGC	Respiratory	MD	≤1
							Respiratory	MD	≥32
11	M	80	2021 (B)	29		CAZ	Urine	MD	1
							Urine	ER	≥32
12	M	90	2021 (B)	14		NA	Respiratory	ER	1
							Respiratory	ER	>8
13	M	52	2021 (B)	31		CST, ETP, CZA	Respiratory	ICU	1
							Respiratory	ICU	16
14	M	53	2022 (C)	19	9 g CI in 24 h	C/T, CST, CZA	Respiratory	ICU	1
							Respiratory	ICU	8
15	M	59	2022 (C)	15	9 g CI in 24 h	C/T, CST, CZA, FOF	Respiratory	ICU	1
							Respiratory	ICU	>8
16	M	38	2022 (C)	32		CST, FDC	Respiratory	ICU	1
							CVC tip	ICU	>8
17	M	49	2022 (C)	19		NA	Respiratory	ER	4
							Respiratory	ER	≥32
18	M	38	2022 (C)	8		TZP, CST, FDC, CZA, MVB	Blood	ICU	≤1
							Respiratory	ICU	>8
19	M	65	2022 (C)	35		CZA, MEM, CST, FDC	Respiratory	ICU	≤1
							Respiratory	ICU	8
20	M	68	2022 (C)	19	3 g every 3 h	CRO, C/T	Urine	MD	0.5
							Respiratory	MD	≥32
21	F	76	2022 (C)	14		MEM, TGC, TZP	Urine	MD	1
							Urine	MD	8
22	F	57	2022 (C)	28		CRO, TZP	Blood	ICU	≤1
							Respiratory	ICU	>8

The first value of minimal inhibitory concentration (MIC) for each patient refers to the C/T-S isolate, while the second value refers to the C/T-R isolate. C/T, ceftolozane/tazobactam; CI, continuous infusion; CRO, ceftriaxone; CST, colistin; CZA, ceftazidime/avibactam; ER, emergency room; ETP, ertapenem; F, female; FDC, cefiderocol; FEP, cefepime; FOF, fosfomycin; g, gram; ICU, Intensive Care Unit; M, male; MD, Medical Department; MEM, meropenem; MVB, meropenem/vaborbactam; NA, not available; SD, Surgery Department; TGC, tigecycline; TZP, piperacillin/tazobactam.

## 4 Discussion

The main result of our study is a statistically significant reduction of the C/T resistance rate in *P. aeruginosa* when the antibiotic was no longer available, followed by a subsequent increase when the antibiotic was reintroduced. However, the analysis of antibiotic consumption and the similar resistance rates observed for other antibiotic molecules, do not allow us to exclude the role of antibiotic stewardship measures on the shift in antibiotic susceptibility for both C/T and other antibiotics. Furthermore, the change in C/T susceptibility in 22 patients with a C/T-resistant isolate after a C/T-susceptible one, the majority of which not exposed to C/T treatment, demonstrates that other antibiotic molecules could induce or modulate C/T resistance.

The maintenance of resistance involves a fitness cost for bacteria, often recovered in the absence of the selective pressure operated by the drug. It has been hypothesized, indeed, that a reduced selective pressure due to lack of antibiotic use would lead susceptible bacteria to outcompete resistant bacteria (Andersson and Hughes, 2010); however, the resistance reversibility has been considered in several studies to be too slow to achieve and difficult to maintain to be of clinical relevance. Additionally, even in the absence of the drug, unexpressed resistance genes can remain in the bacterial population (Baym et al., 2016; Melnyk et al., 2015). After antibiotic use discontinuation, if a percentage of resistant bacteria remains in the population, the resistance rate will rise again when the antibiotic is reintroduced (De Gelder et al., 2004).

Indeed, our study shows a reduction in C/T resistance rate when the drug was not available (25.1% in Period A vs. 5.3% in Period B), supporting the hypothesis of a possible resistance reversion linked with the withdrawn of the drug selective pressure. An increasing trend in C/T resistance after the drug reintroduction (10.0% in Period C) was observed, warning about a return of resistance, at a much faster pace than the original decline, as also reported in the literature (De Gelder et al., 2004; Levin et al., 1997; Austin et al., 1999; Heinemann et al., 2000).

Among the three study periods, we observed an overall 13% resistance rate to C/T, which is higher than reported in European and American data (Karlowsky et al., 2024; Fournier et al., 2021). Moreover, the overall resistance rate observed in our bloodstream infections was higher (16% vs. 4%) than reported in other Italian series (Bianco et al., 2022). Finally, a higher resistance rate to C/T is reported among MDR *P. aeruginosa* both globally (22–31%) and in our country (15–32%), although the signal antibiotics chosen to define the MDR condition vary across studies (Karlowsky et al., 2024; Fournier et al., 2021; Del Barrio-Tofiño et al., 2017; Bianco et al., 2022; De Pascale et al., 2025). In our series, we observed an incidence of resistance to C/T of 43% among carbapenem-resistant strains and 3% among carbapenem-susceptible strains. Interestingly, a Spanish report described 150 extensively drug-resistant *P. aeruginosa* strains where C/T resistance depends mostly on horizontally acquired carbapenemases AmpC overexpression, efflux pumps and OprD inactivation; C/T resistance was not detected in carbapenemase-negative isolates, in agreement with sequencing data showing the absence of ampC mutations (Del Barrio-Tofiño et al., 2017).

Moreover, we observed in our population 22 patients with a C/T-resistant isolate after a previous C/T-susceptible one; only 4 patients among them had received a previous C/T treatment but all were exposed to different antibiotics during hospitalization. This accounts for the complexity of *P. aeruginosa* genome and the high level of resistance that it can develop, due to multiple different mechanisms.

*Pseudomonas aeruginosa* isolates collected in this study were also analyzed for the antimicrobial susceptibility to other drugs: the lowest resistance rate (2.0%) was demonstrated towards colistin followed by amikacin (6.6%), while piperacillin/tazobactam had the highest (29.0%). Among *β*-lactams, we observed a resistance rate similar to the available literature data (Torrens et al., 2022), except for imipenem, C/T and ceftazidime/avibactam which had a lower resistance rate in our series (22.6% vs. 48.0, 12.4% vs. 23.4 and 10.5% vs. 21.4% respectively). These data matched with the consumption of antibiotics, being imipenem, C/T and ceftazidime/avibactam among the least used in our hospital in the three observation periods, according to the DDDs per 100 bed-days per year. Interestingly, we observed a noticeably lower incidence of DTR than European data (Torrens et al., 2022) (1.6% vs. 13.2%), but comparable to the American data (Kadri et al., 2018) (1.6% vs. 2.1%).

Several factors could have been implied in the change in antibiotic susceptibility observed in the other antimicrobials along with C/T. First, being C/T a β-lactam antibiotic, antimicrobial class resistance could be implied, explaining the observed resistance rate of piperacillin/tazobactam, cephalosporin and carbapenem. Production of carbapenemases could be the main resistance mechanism involved, often encoded by plasmids, integrons or other mobile genetics elements, which alter the efficacy of many β-lactams and frequently carry additional resistance determinants, responsible for the lack of efficacy of fluoroquinolones or aminoglycosides (Tenover et al., 2022). Moreover, *in vitro* studies demonstrate indeed an increased resistance development against C/T after meropenem pre-exposure, as a result of stress exposure or molecular level mutations conferring cross-resistance (Fouad et al., 2023).

Additionally, it is interesting to observe that Period A includes two years (2019 and 2020) in which C/T was available, however, 2020 was characterized by the beginning of the Coronavirus Disease-2019 (COVID-19) emergency in our country. Several studies have reported indeed a spread of MDR infection during COVID-19, due to the profound modifications of the healthcare system required by the emergency (García-Meniño et al., 2021; Cantón et al., 2020; Tiri et al., 2020; O’Toole, 2021; Mędrzycka-Dąbrowska et al., 2021), that could have contributed to a change also in our hospital epidemiology. Finally, in 2021 an antimicrobial stewardship (AMS) program was started in our hospital, and this could have contributed to the reduction of resistance rates observed not only for C/T but also for other antimicrobials. Considering the DDD per 100 bed-days per year, we found a similar decreasing trend for both the consumption and the resistance rates of meropenem, ciprofloxacin and colistin. The implementation of the AMS program further influenced antibiotic consumption, in terms of appropriateness and duration of treatments. These elements could explain the reduction of the resistance rates of *P. aeruginosa* towards several antibiotics, from period A to period B, and the persistence of lower resistance rates in period C.

The present study has several limitations, considering the retrospective and monocentric design of the study, results are difficult to generalize. Moreover, no genetic analyses were conducted to assess the potential mechanisms of resistance underlying the observed phenotypic resistance pattern. We hypothesize a role of carbapenemases in many *P. aeruginosa* strains observed, however, we have insufficient data, considering that immunochromatographic tests were not routinely performed in all isolates and that cannot detect all carbapenemases. Finally, a phylogenetic analysis of resistant isolates was not performed to assess the impact of intra-hospital clonal spread.

The unavailability of Zerbaxa® for more than a year created the conditions to analyze the practical application of the theory of fitness cost to maintain resistance. Our data support this hypothesis, showing a consistent reduction of C/T resistance when the antibiotic was not available. However, if a small percentage of resistant bacteria remain in the population, the resistance rate will return high with the reintroduction of the antibiotic. The same mechanism was observed also for other antibiotics, whose consumption was reduced after the implementation of an antibiotic stewardship program in our hospital. Therefore, continuous monitoring of antibiotic use and evolving resistance is essential to properly use our armamentarium against the threat of antimicrobial resistance.

## Data Availability

The raw data supporting the conclusions of this article will be made available by the authors, without undue reservation.

## Author’s note

Data were presented at IDWeek 2023; Oct. 11–15, 2023; Boston, MA.
